# Poly(N-isopropylacrylamide)-Based Thermoresponsive Composite Hydrogels for Biomedical Applications

**DOI:** 10.3390/polym12030580

**Published:** 2020-03-05

**Authors:** Xiaomin Xu, Yang Liu, Wenbo Fu, Mingyu Yao, Zhen Ding, Jiaming Xuan, Dongxiang Li, Shengjie Wang, Yongqing Xia, Meiwen Cao

**Affiliations:** 1 State Key Laboratory of Heavy Oil Processing and Centre for Bioengineering and Biotechnology, University of Petroleum (East China), Qingdao 266580, China; xiaominxu719@163.com (X.X.); yangliu8817@163.com (Y.L.); 1803060126@s.upc.edu.cn (M.Y.); dingzhenshengwu@163.com (Z.D.); mr_xuanjam@163.com (J.X.); sjwang@upc.edu.cn (S.W.); xiayq@upc.edu.cn (Y.X.); 2 Heze Key Laboratory of Water Pollution Treatment, Heze Vocational College, Heze 274000, China; wenbo8109@163.com; 3 Shandong Key Laboratory of Biochemical Analysis, College of Chemistry and Molecular Engineering, Qingdao University of Science and Technology, Qingdao 266042, China; lidx@qust.edu.cn

**Keywords:** poly(N-isopropylacrylamide) (PNIPAM), hydrogels, thermosensitivity, biomedical applications, mechanical strength

## Abstract

Poly(N-isopropylacrylamide) (PNIPAM)-based thermosensitive hydrogels demonstrate great potential in biomedical applications. However, they have inherent drawbacks such as low mechanical strength, limited drug loading capacity and low biodegradability. Formulating PNIPAM with other functional components to form composited hydrogels is an effective strategy to make up for these deficiencies, which can greatly benefit their practical applications. This review seeks to provide a comprehensive observation about the PNIPAM-based composite hydrogels for biomedical applications so as to guide related research. It covers the general principles from the materials choice to the hybridization strategies as well as the performance improvement by focusing on several application areas including drug delivery, tissue engineering and wound dressing. The most effective strategies include incorporation of functional inorganic nanoparticles or self-assembled structures to give composite hydrogels and linking PNIPAM with other polymer blocks of unique properties to produce copolymeric hydrogels, which can improve the properties of the hydrogels by enhancing the mechanical strength, giving higher biocompatibility and biodegradability, introducing multi-stimuli responsibility, enabling higher drug loading capacity as well as controlled release. These aspects will be of great help for promoting the development of PNIPAM-based composite materials for biomedical applications.

## 1. Introduction

Hydrogels are based on using hydrophobic and hydrophilic components to form three-dimensional (3D) network structures under physical and/or chemical interactions, which can imbibe a large amount of water molecules and freeze their movements [1,2,3,4]. In recent years, the development of hydrogels for applications in biomedical areas has aroused great interest from researchers [5]. One of the important types of hydrogels is the stimuli-responsive hydrogels, also called “smart” or “intelligent” hydrogels [6]. These hydrogels can respond to external stimuli to give a reversible volume change or sol-gel phase transition [7,8]. Such a characteristic very effectively mimics the sensitivity and responsibility of biomacromolecules at the in vivo microenvironments [6], which is of superior advantage in developing novel biomedical materials for specific applications [9,10].

Smart hydrogels can respond to a variety of external stimuli such as pH, temperature, light, electric field or ionic strength [3,11,12,13]. Thermoresponsive hydrogels are one of the most widely used smart hydrogels that can be stimulated by a slight change in temperature to give a significant swelling/deswelling process and lead to phase transition [5,14,15]. Basically, thermoresponsive hydrogels can be classified into two categories. One is the hydrogels that have a lower critical solution temperature (LCST). For these hydrogels, the polymer molecules are highly hydrated and dissolve well to give a solution state at lower temperatures [14,16]. However, upon heating the polymer molecules will have a decrease in solubility and aggregate to give a phase transition [17]. In contrast, another type of thermoresponsive hydrogels is the ones that have an upper critical solution temperature (UCST) [9,18]. These hydrogels are in the gelling state below a certain temperature and are soluble above it.

A thermosensitive polymer usually possesses both hydrophilic and hydrophobic groups [19]. Interactions between these groups and the solvent molecules play dominant roles in determining the temperature-responsive behaviors [13,18,20]. Taking the LCST polymers for example, hydrogen bonds can be formed between their hydrophilic groups and water molecules at temperatures below the LCST [21,22]. The highly hydrated polymer molecules can be well dissolved in water at the molecular level [23]. However, at temperatures above the LCST, hydrophobic interaction between the hydrophobic moieties is strengthened while hydrogen bonding is simultaneously weakened [16,18,24]. As a result, the polymer molecules will aggregate to give a coil-to-globule conformational transition, which consequently results in a major shrinking in volume and to extruding water molecules from the aggregates [25].

Poly(N-isopropylacrylamide) (PNIPAM) is one of the most popular thermosensitive polymers that have attracted a lot of research interest [26]. It is comprised of both hydrophilic amide (–CONH–) groups and hydrophobic isopropyl (–CH(CH_3_)_2_) side chains and has a LCST of 32 °C, which is slightly lower than the body temperature of ~37 °C [27,28,29]. Therefore, PNIPAM solutions take a sol state at room temperature and can be transformed into a gel state when approaching the body temperature [30]. This characteristic makes PNIPAM a very excellent candidate for applications in biomedical areas, for example, as carriers for drug delivery, scaffolds for tissue engineering and skin dressings for wound treatment [31,32]. In these applications, the temperature-sensitivity of PNIPAM can be used to achieve specific aims and functionalities. For drug delivery, the drug molecules can be easily loaded into the hydrogel by directly dissolving the drug in the solution at lower temperature and then increasing the temperature to above the LCST for gelation. Moreover, site-specific drug delivery and controlled drug release can be obtained by maintaining fine control over the temperature. For tissue engineering, the PNIPAM solution can be directly injected into the body and triggered by the body temperature to form a 3D hydrogel, which can repair damaged tissues by maintaining the normal physiological activity of cells and promoting cell proliferation and differentiation [33]. Then for wound dressing, the temperature-responsive reversible sol-to-gel phase transition can also be used to control adhesion of the gel coating on the skin as well as its detachment. In addition to thermosensitivity, PNIPAM also has other properties such as tunable structures and low toxicity, which are also of great advantage for biomedical applications.

However, despite these attractive advantages, PNIPAM hydrogels still possess some shortcomings such as low biodegradability, poor mechanical strength, relatively low drug loading capacity and a burst release of the drug molecules [18,34,35,36], all of which greatly hinder their practical applications [8,12]. In recent years scientists have paid great efforts to improve the performance of PNIPAM hydrogels by making up their deficiencies. One effective strategy to achieve this aim is to produce the PNIPAM-based composite hydrogels by using hybridization with suitable species including inorganic nanoparticles, organic self-assemblies and other polymeric components [37,38]. The composite hydrogels have additional and/or enhanced functionalities such as better biocompatibility, enhanced mechanical strength, tunable temperature-responsibility, multidrug loading capacity, targeted drug delivery as well as controlled drug release [33]. These merits will greatly broaden the biomedical applications of PNIPAM hydrogels.

**Scheme 1:**
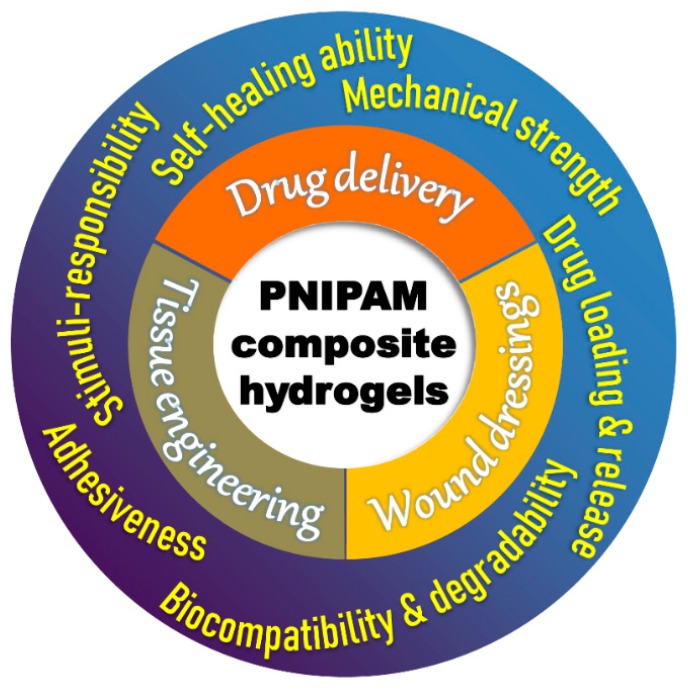
The application areas of Poly(N-isopropylacrylamide) (PNIPAM)-based composite hydrogels as well as the required properties that can be modulated by composite formulation.

This review focuses on the recent studies on PNIPAM-based composite hydrogels for applications in the biomedical areas. It covers the general principles of system design, materials choice and hybridization strategies, as well as the performance improvement of these composite hydrogels. A comprehensive review of the recent studies on the applications of PNIPAM-based composite hydrogels will be given in the following areas, that is, drug delivery, tissue engineering and wound dressings (Scheme 1). We try to give an overview of the general strategies for modulating the properties of hydrogels and demonstrate how to apply these strategies in designing specific PNIPAM-based hydrogels for practical applications.

## 2. General Strategies for Tuning the Properties of the PNIPAM-Based Composite Hydrogels

PNIPAM-based hydrogels have found applications in a vast range of fields, and a specific application will have particular requirements for the properties of the hydrogel. This review focuses on biomedical applications of the PNIPAM-based hydrogels in the areas of drug delivery, tissue engineering and wound dressings. As a result, the following properties of the hydrogels must be considered, which include biocompatibility and biodegradability, drug loading and release properties, mechanical strength, adhesiveness, stimuli-responsibility, a self-healing ability and so on (Scheme 1). In designing an applicable hydrogel system, these properties are usually highly interconnected with each other from the aspects of materials choice to formulating strategy, which usually require a comprehensive consideration of the whole system. Since biosafety is the primary requirement for biomedical applications, the materials chosen for compositing with PNIPAM must have high biocompatibility and low toxicity, regardless of whether natural materials or unnatural ones are used. What comes next is to optimize the properties of the PNIPAM-based hydrogels to meet the requirements of actual applications. Currently there are several common strategies that can be used for modulating the properties of the PNIPAM-based hydrogels, such as co-polymerization with other monomers to produce copolymeric hydrogels and introduction of other polymers or nanoparticles into the PNIPAM hydrogel to form interpenetrating polymer network (IPN) hydrogels or nanocomposite hydrogels [39].

Copolymer formation is one of the most effective strategies used to tune the properties of the polymeric hydrogels. PNIPAM has been copolymerized with other polymeric blocks such as polyethylene glycol (PEG) [34,40,41], dextran [42], chitosan [43,44] and poly(N,N-diethylacrylamide) (PDEAM) [40] to produce copolymeric hydrogels. The new polymeric block can adjust the hydrophilic/hydrophobic balance of PNIPAM to control the thermosensitivity of the resulted hydrogel [45]. A hydrophilic block generally leads to an increased LCST whilst a hydrophobic block gives a decreased LCST. Otherwise, the new polymeric block will change the molecular stiffness and the structure of the hydrogel network, thus modulating the mechanical strength of the copolymeric hydrogel [5,40,41,44]. This can be clearly viewed from the mechanical properties of various PNIPAM-based copolymeric hydrogels as shown in Table 1. Moreover, the biocompatibility and biodegradability of PNIPAM hydrogels can be tuned by choosing another polymeric block with low toxicity and defined degrading routes [46,47,48]. In some cases, the new block may also introduce additional properties to the hydrogels. Recently Li et al. synthesized a tri-block copolymer of DNODN [49]. This tri-block copolymer can form hydrogels that not only maintain a fast thermoresponsive sol-to-gel transition from the PNIPAM block but also have a self-healing ability from self-assembly of the copolymer. Interestingly, the presence of a poly(ethyleneoxide) (PEO) component introduces strong antifouling property to the hydrogel, enabling excellent antifouling performance against nonspecific cell attachment.

Another effective method for modulating the properties of a hydrogel is incorporation of another polymer in the hydrogel matrix to produce IPN hydrogels. IPN hydrogels contain two or more polymers that are independent in structure and interact with each other via physical interactions. Full-IPN is the case when the two polymers both give cross-linked networks, while semi-IPN is produced when a linear polymer is embedded within the first network [53,54]. In the IPN hydrogels, the multicomponent networks can effectively modulate the mechanical property, the swelling/deswelling response as well as the drug loading/release profile of the hydrogel. In the past decades, various polymers have been used to form IPN hydrogels with PNIPAM. Wang et al. [55,56] and Alvarez-Lorenzo et al. [57] have used chitosan to composite with PNIPAM to fabricate either full-IPN hydrogels or semi-IPN hydrogels. For these hydrogels, the properties such as gelation rate, thermoresponsive phase transition, swelling dynamics, drug loading capacity and release profile can be effectively modulated, which are promising for the design and preparation of PNIPAM-based hydrogels for biomedical applications. In the work of Ma et al. [58], polyethyleneimine (PEI) was incorporated into the PNIPAM hydrogel as a general drug carrier. Its introduction not only modifies the structure and size of the pores in the PNIPAM hydrogel but also tunes the water content and water release rate of the hydrogel below the LCST, resulting in a controlled drug release behavior [58]. Shi et al. fabricated thermoresponsive and conductive hybrid hydrogels by in situ crosslinking of phytic acid in PNIPAM matrix to form interpenetrating binary network structures [59]. The architecture of the second polymer in the hydrogel matrix plays an important role in determining the performance of the resulting hybrid material [59].

**Figure 1:**
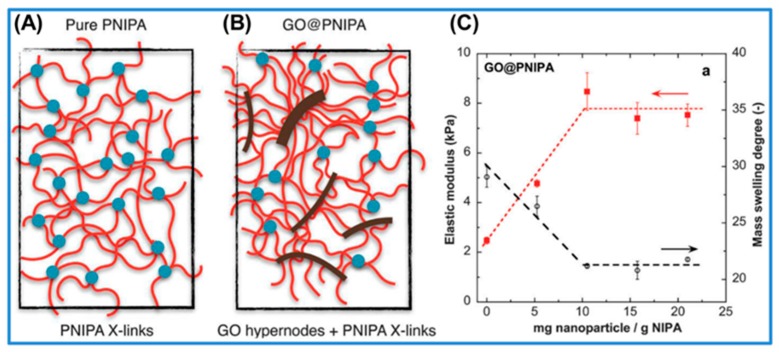
Schematic representation of the structures of (**A**) pure PNIPAM and (**B**) GO/PNIPAM composite gels. (**C**) Equilibrium mass swelling degree in pure water at 20 °C and the elastic modulus of GO/PNIPAM composite gels. Adapted with permission from Ref. [72]. Copyright (2016) The Royal Society of Chemistry. Note that in the source article, PNIPAM is referred to as PNIPA.

In recent years, nanocomposite hydrogels have acquired particular interests. The nanoparticles (NPs) can be incorporated into the hydrogels either by the direct addition of the as-synthesized NPs or by a reaction within the hydrogel to produce NPs in situ or by mixing pre-made nanoparticles with a hydrogel precursor followed by gelation. These nanocomposite hydrogels have a higher degree of chemical or physical complexity and the polymers and nanoparticles can work synergistically to assure tailored characteristics and functionality [60]. Currently many inorganic and organic NPs such as gold NPs [6,60,61], silver NPs [62], carbon nanotubes [63,64], graphene oxide (GO) nanosheets [65,66,67,68,69], clays [35,70] and superparamagnetic iron oxide NPs [71] have been incorporated within PNIPAM hydrogels. These NPs can serve as functional nanofillers to modulate the properties of the hydrogels from various aspects. First, they can mechanically reinforce the hydrogel network and tune the elasticity of the hydrogel. For example, two-dimensional GO nanosheets [65,68,72,73,74] or nano-clays [35,70] have been incorporated within PNIPAM hydrogels, thereby effectively modulating their mechanical properties. In the work of Berke et al. [72], GO platelets were incorporated into the PNIPAM network by using chain nucleation, which resulted in a hypernodal structure of the PNIPAM gel (Figure 1). Increase of the GO content can enhance the elastic modulus and decrease the swelling degree. More examples are presented in Table 2 to show that the NPs incorporation can effectively modulate the mechanical strength of the PNIPAM hydrogels. Moreover, the filler materials can also introduce external stimuli such as light, temperature, pH, an electric field, and a magnetic field to the PNIPAM hydrogels. These stimuli can be used to induce changes of the hydrogel structure as well as to control the drug release. For example, by introducing inorganic gold nanorods (AuNRs) [60] or organic polydopamine nanoparticles (PDA-NPs) [75] and GO sheets [76] with excellent photothermal conversion efficiency into the PNIPAM network, the resulted composite hydrogels show dual thermal and optical responsiveness, which can give phase transition and volume changes under near infrared (NIR) laser irradiation or have a NIR-assisted healing ability. Superparamagnetic iron oxide NPs have also been incorporated into PNIPAM-based hydrogels to enable a magnetic response, which can achieve drug release through remote control with an oscillating magnetic field [71]. Moreover, by adjusting the size of the nanofillers as well as their physical/chemical interactions with the polymeric network, the phase transition temperature of the hydrogel and the responsive speed can also be effectively modulated [73,74,76]. Very recently Czakkel et al. gave a comprehensive review about the effect of graphene-derivatives on the responsivity of PNIPAM-based nanocomposites [65].

Moreover, there are also other strategies that can be used to formulate PNIPAM into double network hydrogels, slide ring hydrogels as well as self-assembled hydrogels, which can effectively modulate the properties of PNIPAM-based hydrogels so as to expand their applications. Some comprehensive review papers on the related topics are available [5,15,18,20] and we will not provide an exhaustive discussion of the topic here.

## 3. PNIPAM-Based Composite Hydrogels for Drug Delivery

With the development of modern biomedicine, targeted therapy and sustained drug release have become very attractive strategies due to their advantages in reducing cytotoxicity, shortening drug cycling period, improving curative effect and so on [16,80,81,82,83,84,85,86]. PNIPAM hydrogel is a promising material for drug delivery that can encapsulate drugs easily and give a temperature-dependent drug release in a slow and linear profile [9,87,88,89]. Recently, PNIPAM-based composite hydrogels have been developed for applications of controlled drug delivery systems, which can overcome the drawbacks of the conventional drug delivery systems by enhancing drug solubility, sustained release time and reducing side effects [14,90,91].

**Figure 2:**
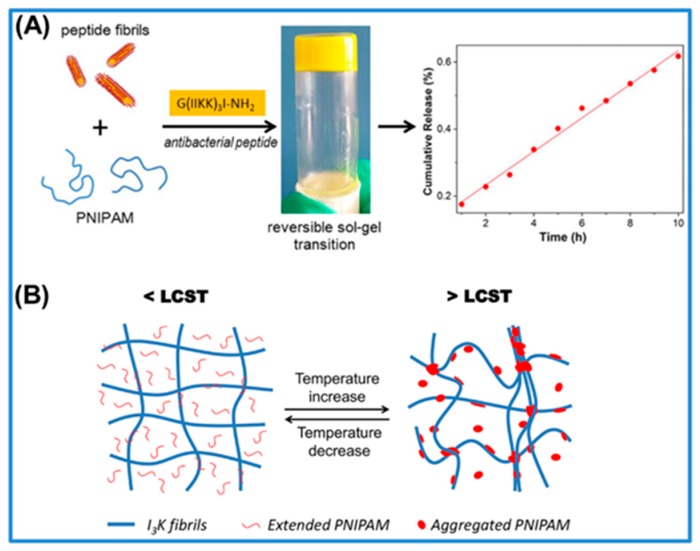
(**A**) Preparation of the thermoreversible peptide/PNIPAM mixed hydrogels and loading of antibacterial peptide G(IIKK)_3_I-NH_2_ for controlled release. (**B**) Schematic diagrams of the proposed states of the I_3_K/PNIPAM networks at temperature either below or above the PNIPAM LCST. Adapted with permission from Ref. [92]. Copyright (2019) American Chemical Society.

Pure PNIPAM hydrogels usually have a limited drug loading ability and can hardly realize a sustained release of drugs due to simple physical interaction forces in the hydrogels, which greatly hinder their applications in drug delivery. Our group has fabricated a hybrid hydrogel by mixing a short peptide of I_3_K with PNIPAM (Figure 2A) [92]. In this system, I_3_K self-assembled fibrils entangle with PNIPAM to form a 3D hydrogel network upon temperature increase to above 33 °C. This sol-gel process is reversible with temperature change because it is driven by physical interactions including hydrogen bonding, hydrophobic interaction and steric hindrance (Figure 2B) [92,93]. Antibacterial peptide G(IIKK)_3_I-NH_2_ was used as a model drug, which can be easily encapsulated in the composite hydrogel by directly adding into the I_3_K/PNIPAM mixed solution at a lower temperature and then increasing the temperature to above 33 °C for gelation. By putting this drug-loaded hydrogel in water at a high temperature, G(IIKK)_3_I-NH_2_ can be released from the hydrogel in a sustained and linear manner. Therefore, this composite system takes advantages of the temperature-responsive phase transition of PNIPAM and the drug loading ability of peptide fibrils to produce a novel hydrogel with thermoreversibility and controlled drug release, which is highly promising for practical drug delivery applications.

The mechanical properties of hydrogels play crucial roles in their biomedical applications. However, poor mechanical strength is one of the drawbacks of PNIPAM hydrogels that greatly restricts their applications. The IPN hydrogels provide an effective strategy for enhancing the mechanical strength of PNIPAM-based hydrogels. Wei et al. have introduced salecan, a kind of natural and hydrophilic polysaccharide, into the network of PNIPAM hydrogels through free radical polymerization [22]. The resulted semi-IPN hydrogels showed enhanced mechanical strength and excellent biocompatibility. Moreover, the semi-interpenetrating structure gave the hydrogels better biodegradability. Similarly, Kim et al. combined PNIPAM hydrogels with hyaluronic acid, a linear polysaccharide with high affinity to water, to form an IPN hydrogel with highly enhanced mechanical strength [27]. The IPN also showed dual pH- and temperature-sensitivity and was used for transdermal drug delivery. Luteolin was successfully encapsulated in the IPN, which can then be released in a sustained manner to alleviate psoriasis. These examples all demonstrate that incorporation of hydrophilic polymers into the PNIPAM hydrogels can greatly enhance the mechanical strength and introduce additional functionalities.

**Figure 3:**
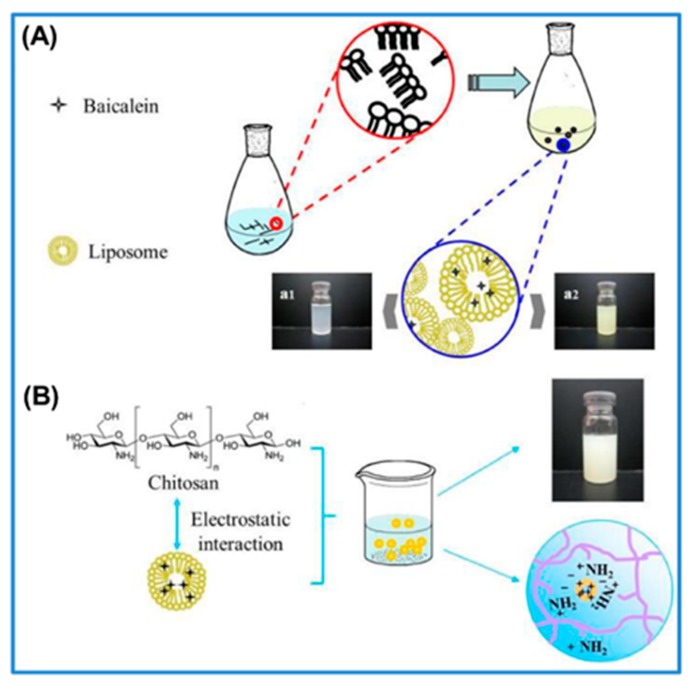
(**A**) Formation of (a) baicaleinliposome (BL) cystic structures and (**B**) chitosan-coated baicalein liposomes. Adapted with permission from Ref. [94]. Copyright (2018) John Wiley and Sons Ltd.

Basically, PNIPAM hydrogels cannot load hydrophobic drugs efficiently due to the absence of hydrophobic binding sites. Feng et al. embedded nano-liposome micelles in poly[(N-isopropylacrylamide)-co-chitosan] (PNIPAM-co-CS) or poly[(N-isopropylacrylamide)-co-(sodium alginate)] (PNIPAM-co-SA) hydrogels to form composite systems [94]. The liposome micelles can encapsulate hydrophobic species in their inner cores and thus greatly enhance the loading capacity for hydrophobic drugs (Figure 3A). In the work baicalein was used as a model drug to be loaded in the composite hydrogel. The micellar encapsulation of baicalein not only enhanced the loading amount but also protected it from oxidization. Moreover, by further involving chitosan component in the composite hydrogel, chitosan can interact with liposomes through electrostatic interaction and the whole system can give a controlled and sustained release of baicalein with both pH and thermal responses (Figure 3B).

How the drug is loaded into the hydrogel and released from it are very crucial in achieving desirable therapeutic outcomes. It is the usual case that a required duration of drug availability and a well-controlled release profile are needed for a specific biomedical treatment. A variety of nanoparticles including carbon-based nanoparticles, polymeric nanoparticles, inorganic/ceramic nanoparticles and metallic nanoparticles can be introduced into PNIPAM hydrogels through physical or chemical cross-linking [69,95,96]. The obtained nanocomposite hydrogels can possess improved drug loading efficacy and a tailored drug release profile that is superior to that of pure PNIPAM hydrogels [97]. For instance, Pan et al. modified the graphene sheets with PNIPAM via click chemistry to obtain the PNIPAM-grafted graphene sheets (PNIPAM-GS) with biocompatibility and thermosensitivity [69]. The superhydrophobic and superoleophilic properties of graphene enable the PNIPAM-GS to load aromatic and water-insoluble drugs (e.g., camptothecin) via van der Waals interaction with a superior loading capacity. The loaded drug can be released in a controlled and sustained manner. In another work, Brunella et al. grafted PNIPAM to mesoporous silica nanoparticles (MSNs) to produce a composite drug carrier [98]. In this system, MSNs can host pharmaceutical compounds and the grafted PNIPAM copolymer plays the role of a gatekeeper, which can realize the temperature-regulated on-off release of drugs from the MSNs’ pores.

The biodegradability of PNIPAM-based hydrogels is less studied than their other properties, however, lack of biodegradability is a significant drawback of them for some practical applications. It may lead to an incomplete release of drugs in drug delivery applications. For tissue engineering, a secondary surgical process will be avoided by using degradable scaffolds that can be degraded via metabolism in the body and then discharged outside. Das et al. incorporated dextrin, a natural polysaccharide that can be easily degraded by amylase, into PNIPAM hydrogel through free radical polymerization to form an oral drug carrier. This delivery system turns out to be a potential dual drug carrier for controlled delivery of ornidazole and ciprofloxacin with good biodegradability [46].

Injectable hydrogels are one kind of novel biomedical materials that keep fluid state outside body and transform into gels after injection into body [15,99,100]. Injectable hydrogels are ideal therapeutic materials because they can be loaded with therapeutic agents and form a 3D network in situ at the diseased sites, forming a drug release depot for pharmaceutics [101,102]. Injectable hydrogels usually need external stimuli such as temperature or pH to trigger the sol-gel phase transition [102,103,104]. The temperature-responsibility of PNIPAM makes it an ideal candidate in formulating injectable hydrogels. One can take advantage of the temperature sensitivity of PNIPAM and the specific properties of other polymers, such as degradability, low toxicity and pH sensitivity, to prepare injectable composite hydrogels for the transport of drugs and cells. These hydrogels can be injected subcutaneously and form drug release depots in situ triggered by body temperature, which have the advantages of not requiring surgery and causing less pain for patients.

PNIPAM-based hydrogels undergo a volume shrink at temperatures above the LCST due to strong intra- and intermolecular hydrophobic interactions [22]. This process will expel water from the hydrogel and accelerates the release of the encapsulated drug to result in a ‘burst effect’ [105], which is unfavorable for the therapeutic process. In order to make up for this drawback, Andrei et al. added dextran to PNIPAM solutions to prepare an injectable composite hydrogel [42]. Compared to pure PNIPAM hydrogel, the composite hydrogel showed greatly enhanced water retention due to the increased hydrophilicity of the hydrogel introduced by dextran. More importantly, the dextran-PNIPAM composite hydrogel can give a controlled and sustained drug release by effectively reducing the ‘burst effect’. In another work, Zhang et al. used carboxymethyl chitosan (CMCS), a derivative of a natural polysaccharide, as the basic material for synthetic hydrogels [100]. They first synthesized a PNIPAM-CMCS copolymer, and then grafted glycidyl methacrylate (GMA) to the copolymer through a ring-opening reaction. The resulted CMCS-PNIPAM-GMA copolymer turned out to be a good injectable hydrogel whose gelation can be triggered by photocrosslinking. By using 5-Fluorouracil and Diclofenac sodium as model drugs, the CMCS-PNIPAM-GMA hydrogels exhibited very good performance in drug delivery with no burst release of drug molecules. In addition, in vitro cell experiments proved that the hydrogels had excellent biocompatibility and negligible toxicity, which are beneficial for biomedical applications. Fathi et al. further prepared dual thermo- and pH-sensitive injectable hydrogels based on chitosan and poly(N-isopropylacrylamide-co-itaconic acid) (PNIPAM-co-IA) [106]. The introduction of itaconic acid endows the PNIPAM hydrogels additional pH sensitivity, greatly widening the application fields. Doxorubicin (DOX) was loaded into the hydrogels for drug release investigation. The results showed that the hydrogels can effectively prolong the release time without a burst release, thereby being suitable for cancer therapy.

In the above section, we present examples on PNIPAM-based hydrogels for drug delivery applications by emphasizing the strategies that can overcome the drawbacks of pure PNIPAM hydrogels. By choosing suitable materials to form composite hydrogels with PNIPAM, the properties and functionalities of the hydrogels can be greatly improved. In these examples, the thermosensitivity of PNIPAM hydrogels is retained, while the introduced components can either act as drug carriers to load more drugs or help to enhance the mechanical strength of the hydrogels and to provide biocompatibility and biodegradability to the systems. These aspects all highlight the enormous potential of PNIPAM-based composite hydrogels for efficient drug encapsulation and release. However, there are still some challenges for drug delivery applications of PNIPAM-based hydrogels. Current biomedical development invokes many needs for novel drug delivery materials. One great demand is to develop novel hydrogel materials that can meet the requirements of severe and complicated disease treatment, for example, cancer therapy, where multidrug delivery to targeted sites and combined therapeutic strategies may be needed. Furthermore, the gene-editing technology such as CRISPR–Cas9 requires efficient delivery of proteins and genes rather than traditional drugs to targeted disease tissues. For these applications, the hydrogels must be designed to protect proteins and genes before reaching the targeted cells and release them easily inside cells. These aspects all provide higher demands for designing PNIPAM-based hydrogels with precise control over the functionalities at the molecular level.

## 4. PNIPAM-Based Composite or Copolymeric Hydrogels for Tissue Engineering

**Figure 4:**
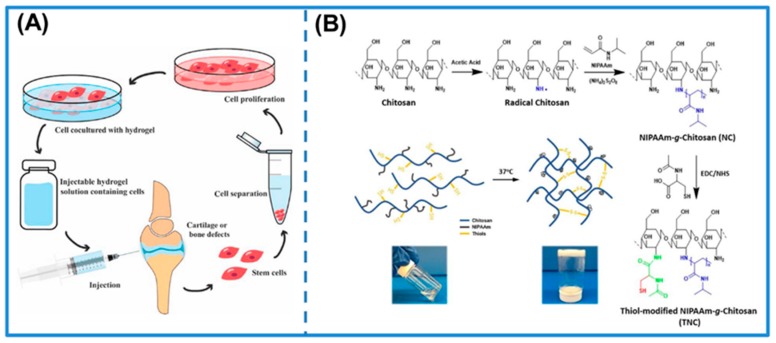
(**A**) Schematic illustration of approaches to make injectable hydrogels for cartilage- and bone tissue-engineering applications. Adapted with permission from Ref. [113]. Copyright (2017) Nature Publishing Group. (**B**) The synthetic routes of NIPAAm-*g*-Chitosan copolymer and its thiolation as well as the temperature-triggered gelation by disulfide bond formation. Adapted with permission from Ref. [44]. Copyright (2018) Elsevier Ltd.

Tissue engineering, also known as regenerative medicine, utilizes the basic principles and methods of cell biology and engineering for researching and developing biological substitutes in order to promote tissue regeneration and recover the morphology and function of tissues or replace the damaged organs [11,13]. In recent years, tissue engineering has developed rapidly, where the cells can first be cultured in the hydrogels to undergo a large number of proliferation and differentiation and then be planted in the body to repair damaged bones, cartilage and other tissues and organs (Figure 4A) [107,108]. Scaffold materials are of great importance for tissue engineering. The scaffolds usually have a 3D structure and require high mechanical strength so as to accommodate the transplanted cells and protect them from damage [105,109]. The scaffolds should also be able to convey sufficient nutrients and growth factors to support cell proliferation and migration as well as to direct cell differentiation into tissues and organs [15,110,111]. Moreover, the ideal scaffold materials must have good biocompatibility, degradability, and non-toxicity [11,109,112,113].

Hydrogels are appealing scaffold materials for tissue engineering by having structural similarity to the extracellular matrix of many tissues [111]. The hydrogel network is filled with a lot of water and the scaffold has a fluid property that allows cells to grow stably in an environment close to physiological conditions [3]. PNIPAM-based hydrogels are highly promising because they are “smart” hydrogels that can respond to temperature change. However, the intrinsic shortcomings of PNIPAM hydrogels, especially low mechanical strength, limited biocompatibility and lack of biodegradability, greatly hinder their applications in tissue engineering [22,101,114]. Choosing suitable species to hybridize with PNIPAM is considered to be one of the most promising strategies to improve the properties and functionalities of PNIPAM-based scaffolds.

The materials used to composite with PNIPAM will modify the properties of the resulted hydrogels by introducing new functionalities and characteristics. It has been demonstrated that the biocompatibility and degradability of PNIPAM hydrogels can be improved by introducing suitable nanomaterials into the system. Cell sheet harvesting (CSH) is an important technique for in tissue engineering. The thermosensitive phase transition of PNIPAM is of great potential for preparing thermo-responsive materials for CSH purposes. However, the native PNIPAM hydrogel is not suitable for CSH due to its low cell attachment. In a recent work, we developed a PNIPAM-based composite hydrogel by incorporating graphene oxide (GO) nanosheets into the system through free radical polymerization with polyethylene glycol dimethacrylate (PEGDA) as a cross-linker [67]. The introduction of GO had little influence on the physical properties of the hydrogel but greatly enhanced its biocompatibility. Compared to the native PNIPAM hydrogels, the cells attached and proliferated better on the GO/PNIPAM composite hydrogels and the intact cell sheets can be harvested from the hydrogels by simply lowering the temperature. Clearly, our work demonstrates the feasibility of incorporating nanoparticles into the PNIPAM hydrogels to improve biocompatibility.

Another effective strategy to regulate the biocompatibility and biodegradability of PNIPAM hydrogels is linking PNIPAM with other polymer blocks of unique properties to produce copolymeric hydrogels that have enhanced biosafety and defined degradation routes introduced by the second polymer block. For example, Spizzirri et al. introduced chitosan, a hydrophilic polysaccharide, into the PNIPAM hydrogel by using free radical grafting, which can impart biocompatibility and biodegradability into the systems [115]. In another work, Mellati et al. also prepared chitosan-*g*-PNIPAM hydrogels to mimic the extracellular matrix of stem cells [48]. These hydrogels can support cell proliferation and give a much larger cell survival ratio than pure PNIPAM hydrogels, indicating significantly improved biocompactibility. The two examples demonstrate the effectiveness of chitosan copolymerization in improving biocompatibility and degradability of the PNIPAM hydrogels. However, these hydrogels still have the problem of low mechanical strength. Wu et al. successfully utilized covalent cross-linking to enhance the mechanical strength of the hydrogels. They incorporated the thiol groups into chitosan, which can be oxidized to form disulfide bonds (S-S) to strengthen the hydrogels (Figure 4B) [44]. The thiol-modified composite hydrogel showed a nine fold increase in the compression modulus. Moreover, these hydrogels showed quite low cytotoxicity and allowed a stable proliferation of mesenchymal cells, fibroblasts and osteoblasts, exhibiting a great potential for being used as carriers to delivery cells in tissue engineering.

The mechanical properties of hydrogels play crucial roles in regulating the interactions between cells and extracellular matrix. To meet the criteria for tissue engineering applications, the mechanical strength of PNIPAM hydrogels can be greatly improved by linking PNIPAM with other polymeric blocks to produce copolymers. Tan et al. synthesized a thermo-sensitive comblike copolymer by connecting carboxylic end-capped PNIPAM and aminated alginate [102]. The resulted composite hydrogels showed good mechanical strength, and the degradation can be controlled by adjusting the grafting ratio of PNIPAM. The hydrogels were successfully used to culture human bone mesenchymal stem cells (hBMSCs), where the cells can maintain good viability and stable proliferation. Polyethylene glycol (PEG) is a non-toxic hydrophilic polymer. Kwon et al. synthesized a series of copolymers by grafting PNIPAM blocks of varied length to a PEG backbone [116]. The copolymers with a high PNIPAM/PEG ratio can produce hydrogels with better mechanical strength and biocompatibility, which were successfully used to immobilize rabbit chondrocytes for culturing. In another work, Ren et al. grafted PNIPAM to a gelatin backbone by using atom transfer radical polymerization (ATRP) [117]. The resulted hydrogel can undergo a thermoresponsive sol-gel transition, which can encapsulate rat hBMSCs into the 3D network conveniently at physiological temperatures for efficient regeneration of bone damage.

Injectable hydrogels can be injected into the patients in an invasive manner under mild conditions, thereby avoiding the painful surgery. Moreover, the hydrogel matrices can support growth of cells for tissue regeneration [34]. Atoufi et al. prepared a complex injectable hydrogel by involving the components of PNIPAM, hyaluronic acid (HA) and chitosan-g-acrylic acid-coated polyactide-co-glycolide (ACH-PLGA) nanoparticles. The system used nanomaterials instead of traditional organic solvents as a crosslinker, which can greatly strengthen the mechanical properties of the hydrogels while avoid toxicity of the organic solvents [118]. Furthermore, the hydrophilic polysaccharide HA is a natural component of an extracellular matrix. Its addition to the hydrogels greatly reduced the syneresis effect of PNIPAM and introduced good biocompatibility, which is highly promising for cartilage tissue engineering.

For application of injectable hydrogels in tissue engineering, the mechanical properties of the hydrogels play crucial roles in determining the therapeutic effects. On the one hand, implanted hydrogels usually suffer from constant external mechanical force after injection, which can lead to deformation or damage to the hydrogels and can shorten the lifespan of the hydrogels [49]. On the other hand, low cell retention and engraftment is a major obstacle for practical applications of injectable hydrogels [119], which is also closely related to the mechanical properties. A weak hydrogel with a shear moduli of <50 Pa is usually required for efficient protection of cell membranes during the injection process [120]. However, the weak hydrogels are often biodegraded or disrupted in a short time, which may not support a long-term cell culturing process [121]. Therefore, it is a great challenge to develop a single hydrogel that can meet these criteria simultaneously. By using a composite formulation, Cai et al. rationally designed a PNIPAM-based hydrogel that can address both of the two aspects, which was termed shear-thinning hydrogel for injectable encapsulation and long-term delivery (SHIELD) [122,123]. The system contains two components. One component is an 8-arm polyethylene glycol (PEG) with one arm conjugated with PNIPAM and the rest of the arms conjugated with proline-rich peptide sequences. The other component is a linear protein copolymer with functional CC43 WW domains and arginine-glycine-aspartic acid (RGD) cell-binding domains. This composite hydrogel can give two different physical crosslinking processes before and after syringe injection. Before injection, molecular recognition takes place between the peptide-PEG copolymer and the recombinant protein to form a weak, physical network ex vivo, which can mechanically protect cells during injection. After injection, thermal phase transition of PNIPAM induces in vivo crosslinking to form a reinforcing network, which can support long-term cell survival within a 3D matrix. These hydrogels can greatly improve transplanted cell retention, which is highly promising for regenerative medicine therapies [123].

In brief, the copolymers synthesized by connecting PNIPAM with other polymeric blocks or functional components not only retain thermosensitivity, but also have highly improved mechanical strength and better biocompatibility and degradability. They can produce hydrogels that mimic very well the structures and properties of extracellular matrix, which can encapsulate cells efficiently and maintain their physiological activity for proliferation and differentiation. Therefore, such hydrogels are highly promising for practical tissue engineering applications. However, there are also many aspects that need to be stressed before clinical translation. One important aspect is to design hydrogel scaffolds that meet the requirements of complicated in vivo applications. First, the performance of the hydrogels should be unaffected by the microenvironments such as body fluids, tissue secrets and joint movement. Second, the implanted hydrogels should have a defined degradation profile that cannot produce toxic byproducts while the degradation speed keeps pace with the tissue regenerative speed. Moreover, the implanted hydrogels for long course tissue repair and regeneration must consider the immune problems, which may require the introduction of specific materials for controlled regenerative immunology.

## 5. PNIPAM-Based Composite Hydrogels for Wound Dressing

Wound treatment is very common in clinical medicine. Traditional materials for wound treatment include gauzes, cotton wools and adhesive bandages. However, these materials are usually considered as passive dressings because they can only provide a simple protection of the wounds and often cause a slow healing process [124]. Moreover, these dressings will dry out and produce a hard plug after absorbing blood or tissue exudate, which adheres tightly to the wounds. Remove of the dressings often leads to secondary injury. Therefore, these dressings are not suitable for treatment of some deep and exuding wounds (e.g., burns, traumatic injuries, surgical incisions and chronic wounds) that have low self-healing ability and usually face problems of wound contamination and infection [125,126]. In recent years, hydrogels have been demonstrated to be ideal wound dressings that have several advantages including good air permeability, the ability to absorb tissue exudates, keeping a moist environment at wound sites and preventing microbial invasion [127]. Besides these merits, PNIPAM hydrogels have some additional advantages for wound dressings. Their thermosensitivity gives chances of using temperature as a simple and effective stimulus to control the properties and functionalities of the dressings. Furthermore, PNIPAM hydrogels have a transparent network, which allows supervision of the healing process. However, despite these advantages, there is still a lot of room for improvement of PNIPAM hydrogels for wound dressing applications, such as enhancing their wound adhesion, optimizing the balance between air permeability and water-retaining property and improving the ability of encapsulation and sustained release of specific drugs to prevent infection. This section briefly summarizes the recent efforts of optimizing the PNPAM-based hydrogels for applications as wound dressings.

Superior mechanical performance is also crucial for wound dressing materials. To compensate for the inherent drawback of poor mechanical strength of PNIPAM hydrogels, Zubik et al. used cellulose nanocrystals (CNCs) to hybrid with PNIPAM via a free radical polymerization to fabricate composite hydrogel properties [79]. CNCs have outstanding mechanical properties and they served as reinforcing agents to enhance the mechanical strength of the composite hydrogels by two mechanisms. One is the covalent cross-link between PNIPAM and the hydroxyl groups of CNCs that reinforces the hydrogels in chemistry. The other is the hydrogen bonds between PNIPAM chains and CNCs that reinforce the hydrogels physically [79].

Grafting the hydrogel onto a supporting material with excellent properties is also an effective method to compensate its inherent drawbacks. Nonwoven fabric is a popular material that has merits of high mechanical strength and convenient handling. Hathaway et al. grafted PNIPAM-co-allylamine (PNIPAM-co-ALA) complex hydrogel on the nonwoven fabric through interactions between the functional groups of ALA and the fabric surface [128]. The resulted composite material had enough mechanical strength to allow for application as bandages. Similarly, Liu et al. also grafted the PNIPAM/polyurethane copolymer hydrogel on the nonwoven fabrics and successfully fabricated the composite wound dressings with high mechanical strength [129].

More significantly, the composite hydrogels with comprehensive properties can be obtained by rational design to incorporate various components. Appropriate water vapor transmission is a very important parameter for an ideal wound dressing to make patients comfortable. For that end, Li et al. first synthesized a PNIPAM/alginate composite hydrogel and then combined it with cotton fabric [127]. The addition of alginate can maintain optimal moist environment and permit good breathability at the wound site. Simultaneously, the combine of alginate and cotton fabric can significantly enhance the mechanical strength of PNIPAM hydrogel. All of these aspects make the composite material an ideal candidate for practical wound treatment.

Silver nanoparticles (AgNPs) have been well demonstrated to be an effective bacteriostatic agent that has a broad-spectrum antimicrobial activity without the development of bacterial resistance, which therefore are highly potential for treatment of wound infection [130]. However, a long term exposure AgNPs in vivo will cause significant cytotoxicity. Formulating AgNPs into the hydrogels is an effective strategy to produce wound dressings with bacteriostatic activities while lowering their cytotoxicity. Qasim et al. embedded AgNPs into PNIPAM-based nanoparticles via in situ reduction [62]. The resulted AgNP-polymeric nanoparticles showed significant bacteriostatic activities against both Gram-negative and Gram-positive bacteria depending on the nanoparticle size and amount of Ag encapsulated in the nanoparticles. Gao et al. synthesized the Ag@PNIPAM nanocomposites via a free radical polymerization [131]. The nanocomposites exhibited high antimicrobial activities against *Staphylococcus aureus* and produced a good therapeutic effect for burn wounds. It is believed that the AgNP-polymeric composites formation improved dispersion of AgNPs and decreased their direct exposure to cells, which reasonably led to an enhancement of the bacteriostatic activities while reducing the cytotoxicity. Such a strategy has also been applied for scald administration, which is a very tough task in clinical therapeutics due to the great challenge of reducing the pain of patients and minimizing the infection of pathogens colonization. Liu et al. designed a PNIPAM-based wound dressing for first aid of scald by integrating PNIPAM hydrogel, sodium polyacrylate and AgNPs into a single system [132]. This dressing possesses hypothermia and antibacterial functions simultaneously, which can be attributed to sodium polyacrylate and AgNPs, respectively. More interestingly, thanks to the thermosensitivity of PNIPAM, the system is “smart” because it can release AgNPs in a reasonable dosage by using temperature as a stimulus to control the release process.

Moreover, through rational design, the PNIPAM hydrogel can be composited with other functional materials to accelerate wound healing by mimicking natural processes. Blacklow et al. have successfully designed a PNIPAM-based hybrid hydrogel by involving PNIPAM, alginate, silver nanoparticles (AgNPs), chitosan and carbodiimide [124]. This hydrogel is an active adhesive dressing (AAD) that mimics embryonic wound closure. It combines the advantages of each component and exhibits thermo-responsive, adhesive, highly-stretchable and antimicrobial functions. Concretely, it has a double-layered network structure: a thermo-sensitive antibacterial matrix formed by the PNIPAM-alginate hybrid network and AgNPs, and a tissue-adhesive surface composed of chitosan and carbodiimide. The incorporation of alginate in PNIPAM hydrogel greatly enhanced the mechanical strength and the mixed AgNPs showed superior effectiveness in preventing wound infection. Besides, when applied in the wound, the strong tissue adhesion achieved by chitosan and carbodiimide-mediated reactions can achieve transfer of the formed contractility to the underlying tissue via phase transition of composite hydrogels. Therefore, the wound dressings can accelerate the healing process in an active manner.

**Figure 5:**
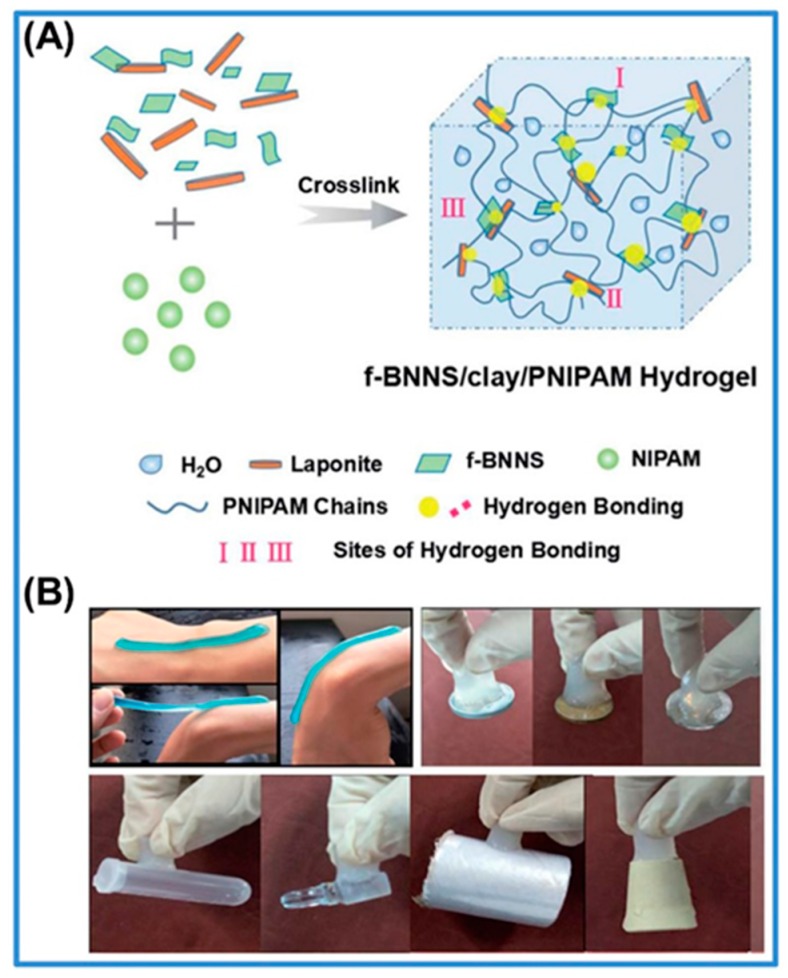
(**A**) Schematic illustration of the formation of the f-BNNS/clay/PNIPAM ternary network hydrogel. (**B**) Hydrogel adhesion on the arm, aluminum, copper, iron, plastics, glasses, polythene and rubbers. Adapted with permission from Ref. [133]. Copyright (2018) The Royal Society of Chemistry.

The adhesiveness of PNIPAM hydrogel to tissue/cell is an important parameter for wound dressing applications. For example, for skin wound treatment, the hydrogels are placed on dynamic surfaces and they need to adhere tightly on skin surfaces and be tough enough to tolerate the skin movements so as to speed up the healing process. However, pure PNIPAM hydrogels usually display a weak adhesion, therefore, it is of great demand to develop PNIPAM-based composite hydrogels with enhanced adhesiveness. Lu et al. introduced polydopamine nanoparticles (PDA-NPs) into PNIPAM network to synthesize the PDA-NPs/PNIPAM composite hydrogel [63]. Since PDA has high adhesion to many substrates through covalent and/or noncovalent interactions, by coating PDA-NPs onto the composite hydrogel surface, the cell/tissue adhesiveness of the hydrogel can be greatly enhanced. The tissue adhesion strength to porcine skin can reach as high as 90 KPa, which can accelerate wound healing. Tong et al. fabricated a peculiar ternary network hydrogel by compositing functionalized-boron nitride nanosheets (f-BNNS) and clay with PNIPAM through physical interactions (Figure 5A) [133]. The composite hydrogel has superior adhesive properties due to interacting with solid surfaces through a hydrogen bond, metal coordination, π-π stacking and cation-π interaction in a synergetic manner. The hydrogel not only can adhere to human skin and be easily peeled off without leaving any residue, but also can adhere tightly to various solid surfaces including aluminum, copper, iron, plastics, glasses, polythene and rubbers (Figure 5B). On the other hand, low adhesion of the hydrogels to tissues or cells is required in some cases. Ohya et al. have conjugated PNIPAM with extracellular matrix-derived biomolecule to produce PNIPAM-grafted hyaluronan (PNIPAM–HA) hydrogels [134]. The adhesion between the PNIPAM-HA covered tissue and adjoining tissues can be greatly reduced due to the highly swollen gel-like structure of HA. Therefore, such hydrogels can prevent post-surgical tissue adhesion and accelerate hemostasis.

To summarize, the PNIPAM-based hydrogels have promising applications as wound dressings. Composite hydrogels formulation can help to optimize the properties of the wound dressings to have the merits of moisture retaining, enhanced adhesiveness, high comfortableness, temperature-triggered precise drug delivery, long time inhibition of infection and allowing supervision of the wound treatment process, which therefore can help to accelerate the healing process of the wounds. This strategy paves the way for application of hydrogels in practical clinical wound administration. Currently the challenging topics for wound dressing applications of the PNIPAM-based hydrogels focus on development of tough and highly adhesive hydrogels that can meet specific clinical requirements. For example, sometimes the inside tissues such as the heart may be injured accidently or by surgery. In this case, the wound dressing should adhere and conform robustly to the tissue surfaces with many body fluids and dynamic movements, which provide exceptional requirements for the superior mechanical strength and adhesiveness of the hydrogel.

## 6. Concluding Remarks and Future Perspectives

To summarize, PNIPAM-based thermoresponsive hydrogels demonstrate great potential in many biomedical areas, from drug delivery to tissue engineering and wound dressings. A specific advantage is that PNIPAM has a LCST of about 32 °C and can be easily triggered by body temperature to give a phase transition, which makes PNIPAM-based hydrogels highly suitable for practical applications. Though PNIPAM hydrogels themselves have the drawbacks of low mechanical strength, limited drug loading capacity, low biodegradability and so on, they can be formulated with other components to form composited hydrogels so as to make up for these deficiencies. Researchers have developed several effective strategies to improve the properties of PNIPAM-based hydrogels, which include incorporating functional inorganic nanoparticles or self-assembled structures into the system either physically or chemically and linking PNIPAM with other polymer blocks of unique properties to produce copolymeric hydrogels. These strategies can successfully enhance the mechanical strength, give higher biocompatibility and biodegradability, introduce multi-stimuli responsibility, or enable multidrug encapsulation and higher drug loading amount as well as controlled release, which therefore greatly promote the practical applications of PNIPAM-based hydrogels.

However, despite being promising, there are still some major concerns and challenges in developing PNIPAM-based hydrogels for biomedical applications. Currently, it is still a great challenge to optimize the properties of the composite hydrogels by choosing suitable hybridizing materials and strategies. The practical biomedical applications usually face complicated conditions and require extremely strict property controls over the composite hydrogel. Therefore, controlled fabrication of PNIPAM-based hydrogels with required properties is of great importance to improve their therapeutic efficacy. Moreover, most state-of-the-art PNIPAM-based hydrogels are still at the stage that is far from clinical practice. For biomedical applications, materials fabrication is only the first step. After that, more preclinical toxicological and pathological studies must be performed to get a detailed understanding and control of the physiological stability, toxicity, immune response, pharmacokinetics as well as biodegradation and elimination of the biomedical materials [80]. Despite this, the PNIPAM-based thermoresponsive hydrogels can obtain immense diversity by hybridizing with other species to produce the composite systems, which are highly promising for practical applications in various biomedical fields [87].

## Figures and Tables

**Table 1:** Mechanical properties of the PNIPAM-based copolymeric hydrogels.

Matrix	Concentration (M) ^a^	Second Polymer Block	Concentration	Mechanical Strength (kPa)	Refs
PNIPAM	7.5	-	-	Y = 200	[50]
PNIPAM	0.22	-	-	CS = 3.8	[51]
PNIPAM	3.31 × 10^−4^	Dextrin	9:1 ^b^	G′ = 8.5	[46]
PNIPAM	0.22	Chitosan/NAC	1:5 ^c^/8:1 ^c^	G′ = 57.27 CS = 11.4	[44]
PNIPAM	0.41	Chitosan	3:20 ^c^	G′ = 0.155	[48]
PNIPAM	0.04	Alg	32:7 ^c^	G′ = 808	[52]

^a^ Based on NIPAM monomer; ^b^ molar ratio of dextrin to PNIPAM; ^c^ weight ratio of the second polymer block to NIPAM; G′: Storage modulus; Y: Young’s modulus; CS: Compressive strength; NAC: N-acetyl-cysteine; Alg:Allyl-substituted alginate.

**Table 2:** Mechanical properties of the PNIPAM-based nanocomposite hydrogels.

Matrix	Concentration (M) ^a^	Nanoparticle	Mechanical Strength (kPa)	Refs
PNIPAM	0.7	silica	Y = 160	[77]
PNIPAM-catechol	4.71	Au	G′ = 710	[78]
PNIPAM	0.88	Clay/PDA	CS = 223	[4]
PNIPAM	0.4	GO	CS = 216	[68]
PNIPAM	1.25	CNC	G′ = 0.55	[79]
PNIPAM	0.24	Clay	TS = 995	[70]

^a^ Based on NIPAM monomer; G′: Storage modulus; Y: Young’s modulus; TS: Tensile strength; CS: Compressive strength.
